# Multiple occurrence of psychomotor retardation and recurrent miscarriages in a family with a submicroscopic reciprocal translocation t(7;17)(p22;p13.2)

**DOI:** 10.1186/s12920-018-0384-4

**Published:** 2018-08-20

**Authors:** Magdalena Pasińska, Ewelina Łazarczyk, Katarzyna Jułga, Magdalena Bartnik-Głaska, Beata Nowakowska, Olga Haus

**Affiliations:** 1 Department of Clinical Genetics, Faculty of Medicine, Collegium Medicum in Bydgoszcz, Nicolaus Copernicus University, Skłodowskiej - Curie 9, 85-094 Bydgoszcz, Poland; 2 Department of Medical Genetics, Institute of Mother and Child, Kasprzaka 17A, 01-211 Warsaw, Poland

**Keywords:** Chromosomal abnormalities, Recurrent miscarriages, Reproductive failure, dup17p13.3 syndrome, Genetic counselling

## Abstract

**Background:**

Balanced reciprocal chromosomal translocations (RCTs) are the ones of the most common structural aberrations in the population, with an incidence of 1:625. RCT carriers usually do not demonstrate changes in phenotype, except when the translocation results in gene interruption. However, these people are at risk of production of unbalanced gametes during meiosis, as a result of various forms of chromosome segregation. This may cause infertility, non-implantation of the embryo, shorter embryo or foetus survival, as well as congenital defects and developmental disorders in children after birth.

The increasing popularity of cytogenetic molecular techniques, such as microarray-based CGH (aCGH), contributed to the improved detection of chromosomal abnormalities in patients with intellectual disability, however, these modern techniques do not allow the identification of the balanced in potential carriers. Therefore, classical chromosome analysis with GTG technique still plays an important role in the identification of balanced rearrangements in every case of procreation failure.

**Case presentation:**

In this article, a family with multiple occurrences of 17p13.3 duplication syndrome in the offspring and multiple miscarriages resulting from carrying of the balanced reciprocal translocation t(7;17)(p22;p13.2) by proband father is presented.

The aCGH diagnostics allowed the identification of an unbalanced fragment responsible for the occurrence of clinical signs in the female patient, while karyotyping and FISH using specific probes allowed the localization of the additional material in the patient chromosomes, and identified the type of this translocation in the carriers.

**Conclusions:**

Identification of a balanced structural aberration in one of the partners allows direct diagnostics for the exclusion or confirmation of genetic imbalance in the foetus via traditional invasive prenatal diagnostics. It is also possible to use an alternative method, Preimplantation Genetic Diagnosis (PGD) after in vitro fertilization, which prevents initiating pregnancy if genetic imbalance is detected in the embryo.

## Background

The incidence of balanced, i.e. reciprocal chromosomal translocations, Robertsonian translocations or inversions, in the general population is 0.7% and increases to 4.8% in couples after two miscarriages and to 5.2% in couples after three miscarriages [1]. In couples in which a pregnancy that ended with stillbirth or birth of a child with malformations occurred, the risk of carrying by one of the partners is higher and can reach 16% [2]. Carrying of balanced aberrations usually does not affect the carrier life span or health status. However, unbalanced gametes can be formed during meiotic divisions, which leads to miscarriages, stillbirths or births of disabled children [1, 3]. Development of new techniques of molecular cytogenetics, i.e. array Comparative Genomic Hybridization (aCGH) and Multiple Ligation-dependent Probe Amplification (MLPA), allowed for analysis of small regions of imbalance and for discovery of new genetic syndromes associated with microdeletions or microduplications that were not previously identified using classical cytogenetic methods [3, 4].

Identification of a balanced structural aberration in one of the partners allows direct diagnostics for the exclusion or confirmation of genetic imbalance in the fetus via traditional invasive prenatal diagnostics. It is also possible to use an alternative method, Preimplantation Genetic Diagnosis (PGD) after in vitro fertilization, which prevents initiating pregnancy if genetic imbalance is detected in an embryo [1, 5].

In this article, a family with multiple occurrences of 17p13.3 duplication syndrome in the offspring and multiple miscarriages resulting from carrying of the balanced reciprocal translocation t(7;17)(p22;p13.2) by proband father is presented.

## Case presentation

An unrelated couple, woman aged 29 and man aged 33 years, were consulted for preconception guidance at the Genetic Outpatient Clinic before their first pregnancy due to the familial history of miscarriages and psychomotor/intellectual delay. Physical examination of both partners did not reveal any phenotypic abnormalities or chronic diseases.

In the man’s family, three out of four of his adult siblings, his father’ brother, and two out of three children of healthy father’s sister, showed intellectual disability. Moreover, his mother had five spontaneous miscarriages in the first trimester of pregnancies.

**Fig. 1:**
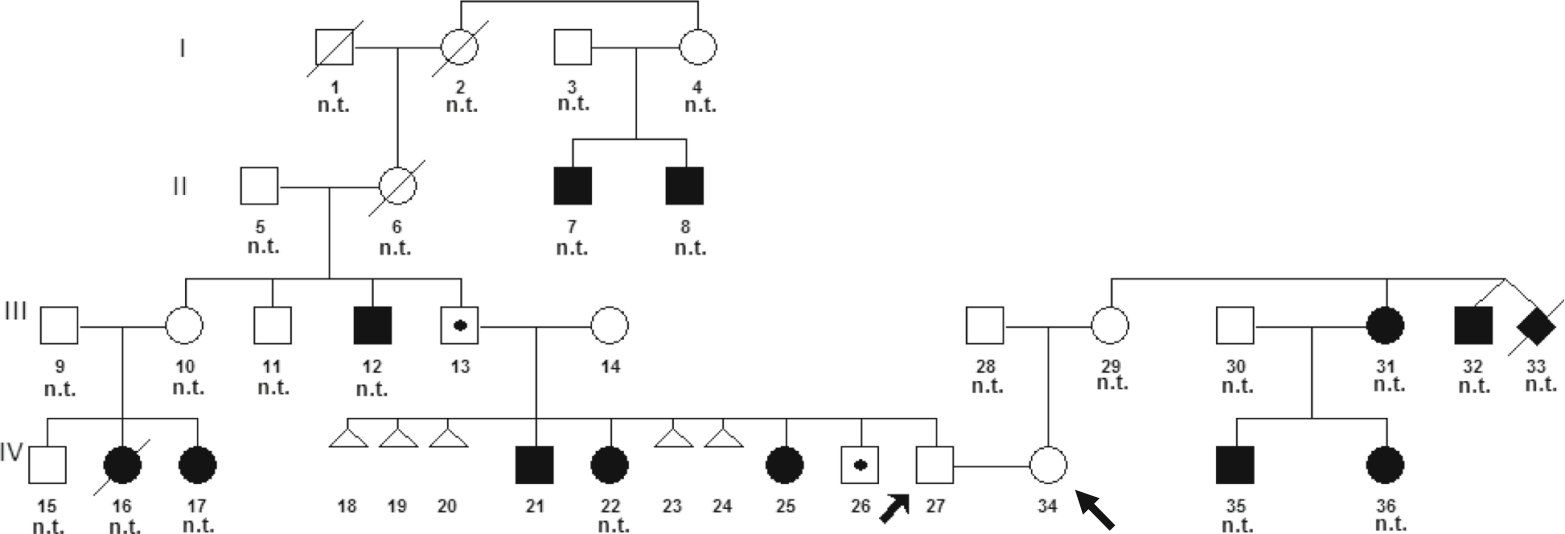
Pedigree of a man’s family, a consulted couple. Legend: - white symbol (square or circle) - a healthy person, a symbol (square or circle) with the black dot - carrier of the balanced translocation t(7;17), black symbol (square or circle) - a person with mental retardation. Triangle – miscarriage, black diamond - intrauterine death, − n.t. - not tested genetically, − arrows - consulted couple

The woman from the consulted couple also had a familial history of intellectual disability; two her siblings were intellectually disabled as well as children of one them. However, the family did not consent to undergo genetic tests (Fig. 1).

Three disabled siblings of the man were consulted in genetic outpatients clinic.

First patient (IV/21) - a man, aged 40 years at the cytogenetic examination, height 176 cm, weight 90 kg, born in the 38th week of pregnancy, weighted 3200 g, obtained Apgar score of 8.

The patient attended a school for children with moderate and severe intellectual disability. At the time of diagnosis he did not read or write, but understood spoken language. Willing to help with household activities.

Second patient (IV/22) - a woman, aged 39 years at the time of cytogenetic examination, height 168 cm, weight 90 kg. Born in the 36th week of pregnancy, weighted 2800 g, obtained Apgar score of 7.

Third patient (IV/25) - a woman, aged 31 years, height 170 cm, weight 70 kg. Born in the 36th week of pregnancy, weighted 2700 g, obtained Apgar score of 7.

In the these three siblings, excessive hypotonia and delayed psychomotor development were found. They started to sit at 14 and walk at 20 months of age. Sleep disorders and very good memory were observed particularly in the female patients, while all three siblings exhibited development of speech comparable to that of their healthy siblings. Currently, their speech is unclear, vocabulary is poor and sentences are simple. In the female patients, premature puberty occurred, at the age of 9 years, followed by hirsutism remaining to date. Bed-wetting was present until 15 years of age. Both female patients graduated from a special school and can read and write.

In all three disabled siblings psychiatric problems started in early childhood, with serious depression during puberty. In all three, dysmorhpic features are present, i.e. triangular face with slight asymmetry, hypertelorism, down-slanting palpebral fissures, broad nasal root and rounded nasal tip, prognathism, high palate, low-set ears and hypodontia. In all, excessive weight is observed, as well as increasing scoliosis of the Th–L segment. All have shortened 3rd, 4th and 5th toes of both feet. All three also demonstrate higher pain threshold, which leads to frequent, deliberately caused self-injuries.

The material for the study was peripheral blood. Blood cells were cultured for 72 h with PHA, with the culture being carried out and harvested routinely. The cytogenetic slides were stained using the GTG technique, which was supplemented with fluorescent in situ hybridization (FISH). The following molecular probes were used: specific for the critical region of Miller–Dieker syndrome and for the critical region of Smith–Magenis syndrome [MD Miller-Dieker LIS (17p13)/Smith-Magenis RAI (17p11) [Kreatech], specific for *TP53* gene (TP53 Deletion Probe, Cytocell), specific for subtelomere of the short arm of chromosome 7 (Subtelomere Specific Probe 7pter, Cytocell) and for subtelomere of the short arm of chromosome 17 (17pter Subtelomere, Leica). The FISH analysis was carried out according to the instructions of manufacturers of the molecular probes used.

GTG analysis was carried out in both partners (IV/27 and IV/34) and in one intellectually disabled sister of the male partner (patient IV/25). Normal karyotypes were found in both partners. In patient IV/25 an addition to the short arm of chromosome 7 was identified by GTG. aCGH examination using a whole-genome oligonucleotide microarray with the mean resolution of 60 kbp revealed a duplication of 4.2 Mbp in chromosome 17 short arm (dup17p13.3p13.2).

Array comparative genomic hybridization (array-CGH) was performed using commercially available array (CytoSure, Constitutional v3 (8x60k), Oxford Gene Technology (OGT), Oxfordshire, UK), according to the manufacturer’s protocol. The CytoSure Interpret Software (OGT) was used for genomic copy-number analysis.

**Fig. 2:**
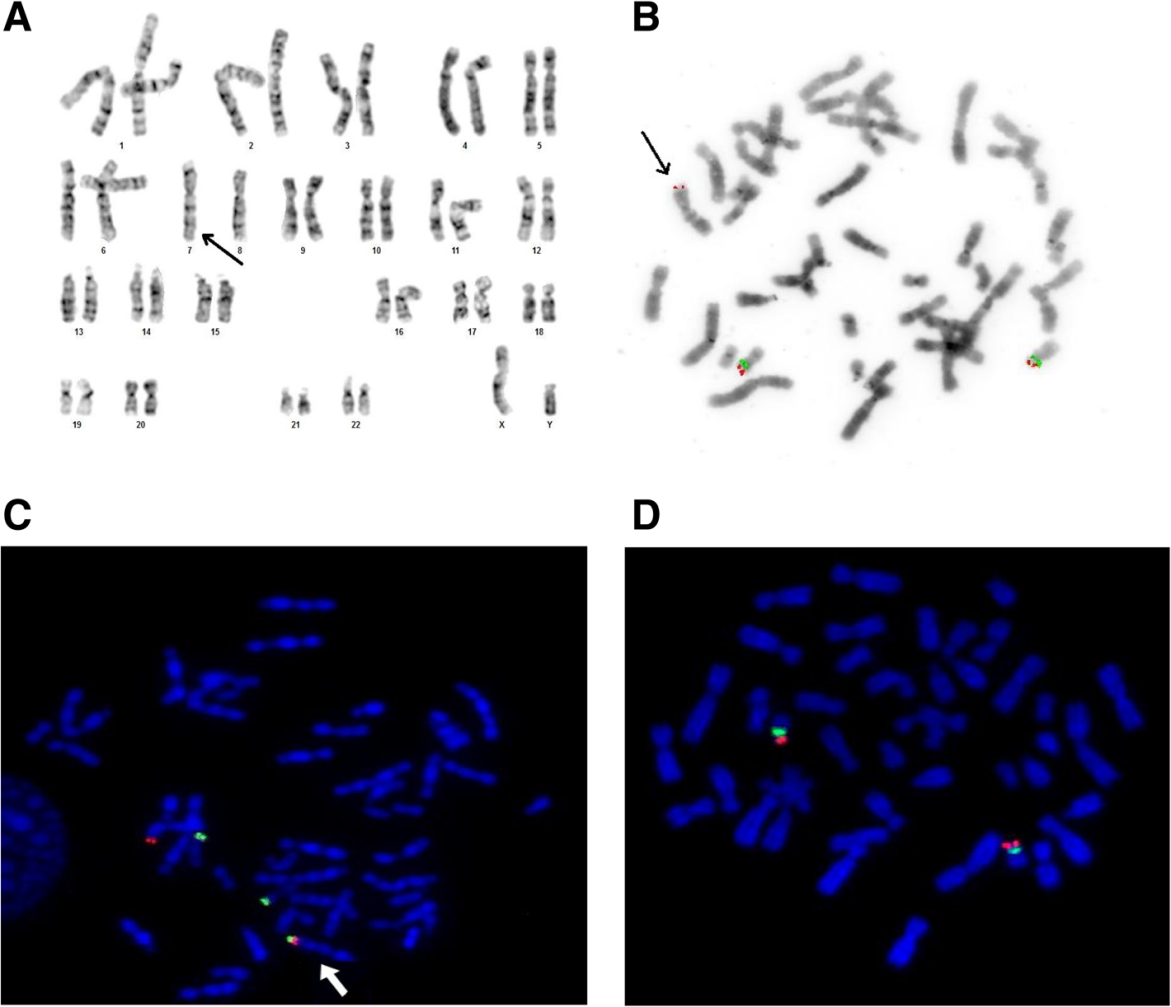
**a** Karyogram of a patient IV/21 with intellectual disability and a karyotype 46,XY,der(7)t(7;17)(p22;p13.2). The arrow indicates the abnormal chromosome 7. **b** Metaphase spread of the patient IV/21 stained using FISH technique with a probe specific for the critical region of Miller–Dieker syndrome (17p13.3—*LIS1*, red) and the critical region of Smith–Magenis syndrome (17p11.2—*RAI1*, green). Both signals are visible on short arms of both chromosomes 17. An additional, third LIS1 signal is visible on chromosome 7 short arm. The arrow indicates abnormal chromosome 7. **c** Metaphase spread of the patient IV/21 stained using FISH technique with a probe specific for the subtelomere of chromosome 7 short arm (red) and a probe specific for the subtelomere of chromosome 17 short arm (green). The arrow indicates abnormal chromosome 7 with two red/green signals from chromosome 7 and chromosome 17 subtelomeres. **d** Metaphase spread of the aforementioned patient (IV/21) stained using FISH technique with a probe specific for *TP53* gene (17p13.1, red) and chromosome 17 centromere (green). These two signals are present on both chromosomes 17

The detected change was confirmed by FISH technique using the probe specific for the critical region of Miller–Diecker syndrome (MDS)(17p13.3—*LIS1*). The analysis revealed three signals of the MDS critical region and two normal signals of the Smith - Magenis syndrome (SMS) critical region (17p11.2—*RAI1*). The additional signal from the *LIS1* (*PAFAH1B1*) gene was located on the short arm of chromosome 7 (7p22), which confirmed the presence of an unbalanced t(7;17) in the patient. Her karyotype was established as 46,XX,der(7)t(7;17)(p22;p13.2) (Fig. 2a, b, c). Additionally, a FISH test with the probe specific for the *TP53* gene (17p13.1) was done, which revealed the presence of two normal copies of *TP53* located on chromosomes 17 (Fig. 2d). The same change of MDS critical region, responsible for 17p13.3 duplication syndrome, was also found in the patient’s disabled brother. The older disabled sister (patient IV/22), presenting similar clinical symptoms, did not consent to genetic testing.

After obtaing the results of aCGH and FISH examinations in disabled patients, cytogenetic preparations of proband, his parents and his healthy brother underwent FISH examination with probes specific for the subtelomeres of chromosomes 7 and 17 short arms and for the critical regions of Miller–Dieker syndrome (*LIS1*) and Smith–Magenis syndrome (*RAI1*), as well as probes specific for *TP53* (17p13.1).

**Fig. 3:**
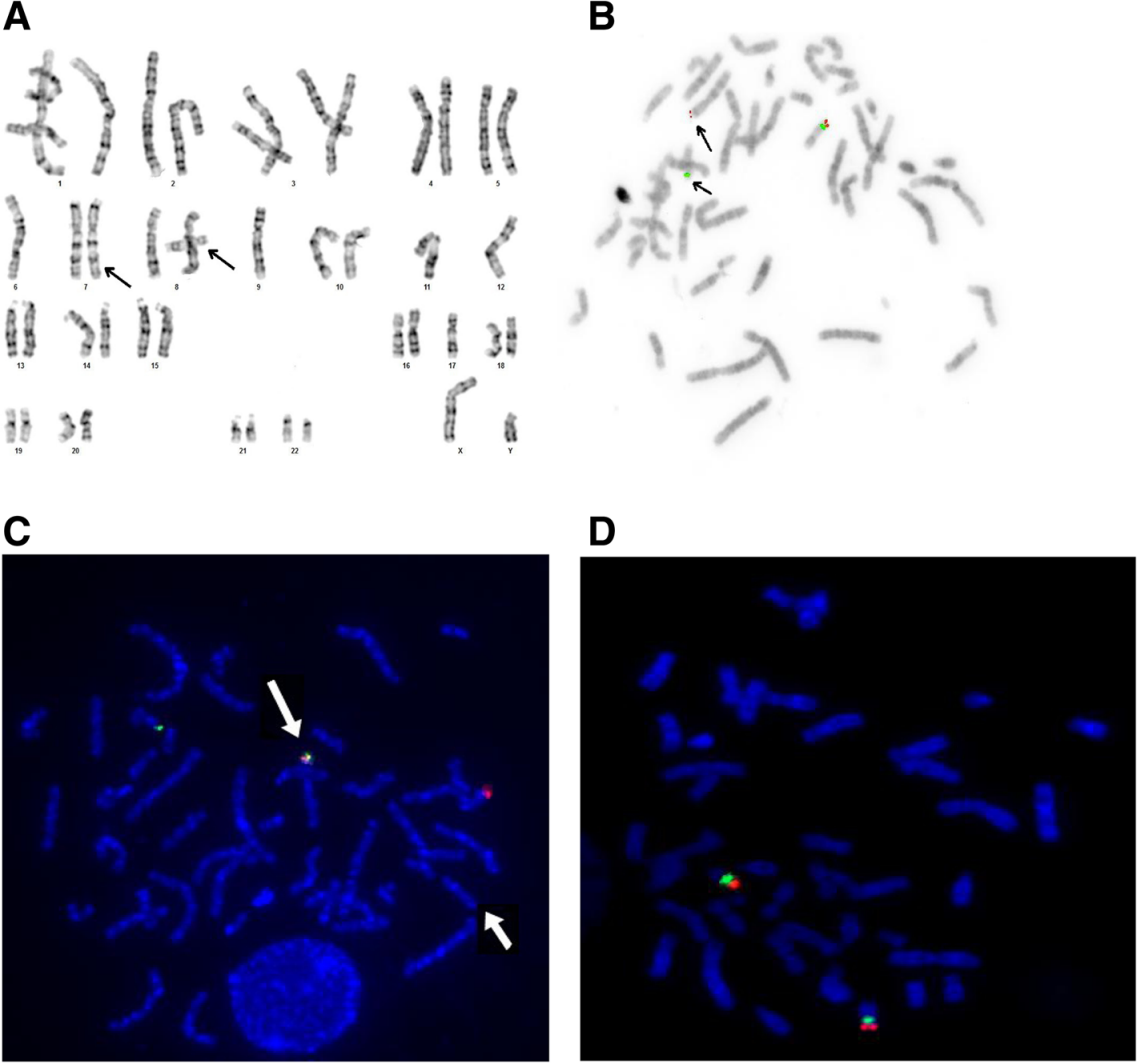
**a** Karyogram of the proband father III/13, carrier of the balanced translocation; t(7;17)(p22;p13.2). The arrows indicate abnormal chromosomes 7 and 17. **b** Metaphase spread of patient III/13 stained by FISH technique with a probe specific for the critical region of Miller–Dieker syndrome (17p13.3—*LIS1*, red) and the critical region of Smith–Magenis syndrome (17p11.2—*RAI1*, green). The arrows indicate the abnormal chromosomes 7 and 17. **c** Metaphase spread of the patient III/13 stained by FISH technique with a probe specific for the subtelomere of chromosome 7 short arm (red) and a probe specific for the subtelomere of chromosome 17 short arm (green). The longer arrow indicates the abnormal chromosome 7 with two signals from chromosome 7 and chromosome 17 subtelomeres. The shouter arrow indicates chromosome 17 without subtelomere signal. **d** Metaphase spread of the patient III/13 stained by FISH technique with a probe specific for the *TP53* gene (17p13.1, red) and chromosome 17 centromere (green). The number and localization of signals are normal

Balanced translocation between chromosomes 7 and 17 was excluded in the proband and confirmed in his father and healthy brother (IV/26)(Fig. 3a, b, c, d).

## Discussion & conclusions

RCTs are the ones of the most common structural aberrations in the population, with an incidence of 1:625 [1, 2, 5]. They arise from breaks of usually two chromosomes and the subsequent reciprocal lossless transfer of genetic material between these chromosomes. RCT carriers usually do not demonstrate changes in phenotype, except when the translocation results in gene disruption [2, 5].

However, these people are at risk of production of unbalanced gametes during meiosis, as a result of various forms of chromosome segregation. This may cause infertility, non-implantation of the embryo, shorter embryo or fetus survival, as well as congenital defects and developmental disorders in children after birth [2, 5].

The probabilities of different unfavorable pregnancy outcomes for carriers of particular RCTs depend strongly on the type, size, and genetic content of unbalanced chromosomal segments involved in RCT [1, 2, 5].

FISH and aCGH tests carried out in the analyzed family allowed the diagnosis of duplication of chromosome 17 short arm covering the region 17p13.3p13.2 of 4.2 Mbp, containing the region of 17p13.3 duplication syndrome (OMIM 613215) and enabled the identification of asymptomatic carriers in the family as well as the exclusion of the carrier status in the proband.

The presence of high density low copy repeats (LCRs) on chromosome 17 short arm promotes the occurrence of submicroscopic rearrangements [6, 7].

Microduplications in 17p13.3 occur in the same gene region that when deleted causes MDS, therefore this region is sometimes referred to as the MDS critical region. Duplications that cause 17p13.3 microduplication syndrome have various underlying mechanisms, various sizes, and include a variety of genes [3, 4, 8].

The following genes are found in17p13.3 chromosome region, according NCBI database; *BHLHA9, YWHAE, MYO1C, INPP5K, PITPNA, SLC43A2, SCARF1, RILP, PRPF8, TLCD2, WDR81, SERPINF2, SERPINF1, SMYD4, RPA1, RTN4RL1, DPH1/OVCA1, OVCA2, HIC1, SMG6, SRR, TSR1, SGSM2, MNT, METTL16, PAFAH1B1* [8] (https://www.ncbi.nlm.nih.gov/gene/). The phenotypes generally associated with microduplications of 17p13.3. include developmental and psychomotor delay, behavioral problems and autism spectrum disorder (ASD), structural brain abnormalitis, and distinct physical features [8].

The deletions and duplications overlapping the *PAFAH1B1* and *YWHAE* genes located in the 17p13.3 region are associated with different clinical phenotypes.

Deletion of only *PAFAH1B1* gene causes isolated lissencephaly, while deletion of both of the aforementioned genes causes Miller–Dieker syndrome [6, 9, 10]. Isolated duplication of the *PAFAH1B1* gene is associated with mild mental disability and hypotonia, while isolated duplication of the *YWHAE* gene causes intellectual disability and autism [7, 10].

Although the duplication identified in the proband (patient IV/25) covered a region of 4.2 Mbp, clinical signs such as impairment of psychomotor development, autistic disturbances, dysmorphic face, syndactyly of the 3rd and 4th toes, hypotonia and obesity indicated phenotypic type I of the 17p13.3 duplication syndrome, as proposed by Bruno et al. [10, 11]. According to this classification, type I is characterized by the presence of duplication of the *YWHAE* gene that has an impact on the development and maturation of neural network. In type II, duplication always comprises the *PAFAH1B1* gene and partially also the *CRK* and *YWHAE* genes. These changes (type II) are associated with hypotonia, disturbances of psychomotor development, dysmorphy, microcephaly and growth cessation [11, 12]. According to Bruno et al., duplication of the *PAFAH1B1* gene with the concurrent duplication or deletion of the *YWHAE* and *CRK* genes leads to a milder neurologic phenotype than duplication of the *PAFAH1B1* gene alone, which may indicate a possible interaction between the *CRK, YWHAE and PAFAH1B1* genes as the key molecular pathways that control the development and migration of cerebral cortex neurons [11–13]. Similarly, Bi et al. suggest that dysmorphic face is present in patients with duplication of the *YWHAE* gene, but not present in patients without its duplication [13]. According to these authors, duplication of the *YWHAE* gene is also associated with macrosomia, but only if the additional material includes the *CRK* gene that participates in the regulation of cell growth and differentiation [11, 13].

Previous studies reported patients with intellectual disability as well as with psychomotor development disorder in whom the dup17p13.3 syndrome was diagnosed [8].

Previous studies showed the connection of the microdplication syndrome 17q13.3 with limb defects and cleft palate [14, 15]. In the studies of Klopocki et al., in 30% of patients with split food malformation with long bone deficiency 17p13.3 microduplications were found, encompassing *BHLHA9* gene [14]. However, the *BHLHA9* is located within chromosome 17p13.3, but immediately outside the MDS critical region [8, 14, 15]. Thus, it can be concluded that the proband (IV/25) carried both genes in double copies.

The proband’s father and healthy brother were diagnosed as carriers of the balanced reciprocal chromosomal translocation t(7;17)(p22;p13.2) associated with the risk of production of gametes with a duplication or deletion of 17p13.3 region. In the case of the proband father, carrying of the translocation resulted in the intellectual disability of three liveborn children and in five miscarriages in his wife. However, due to cytogenetic diagnosis proband’s brother and his partner, who do not have children as yet, and did not suffer from miscarriages, may undergo preimplanatation cytogenetic diagnostics or prenatal cytogenetic diagnostics.

Previous literature reports showed that using new diagnostic techniques such as MLPA, aCGH or NGS allowed for establishing of phenotype-genotype correlations. The use of FISH with commercially available probes to recognize smaller deletions may result in a wrong diagnosis [8, 14, 15].

Our research showes possibilities and difficulties in diagnosing of healthy couple with history of intellectual disability in the family. Whole family involvement and determination of a consulted couple allowed to establish the diagnosis for symptomatic adult sibling.

In standard clinical practice, conventional cytogenetic methods that due to their low resolution do not allow the identification of submicroscopic unbalances, are still frequently used. A more exact characterization of these rearrangements is necessary for the proper genetic diagnosis of a patient and determination of the carrier status and genetic risk in the family members. The increasing popularity of cytogenetic molecular techniques, such as microarray-based CGH (aCGH), contributes to the improved detection in patients with intellectual disability, however, these modern techniques do not allow the identification of the balanced in potential carriers [1, 4, 7]. Therefore, classical chromosome analysis with GTG technique still plays an important role in the identification of balanced rearrangements in every case of procreation failure.

In the case of the consulted couple, family diagnostics was possible after the identification of a small chromosomal abnormality in the proband’s disabled sister (IV/25). The aCGH diagnostics allowed the identification of an unbalanced fragment responsible for the occurrence of clinical signs in the female patient, while karyotyping and FISH using specific probes allowed the localization of the additional material in the patient chromosomes, and identified the type of this translocation in the carriers [1, 2, 5, 8].

## Abbreviations

aCGH: array Comparative Genomic Hybridization
FISH: Fluorescent in situ Hybridization
GTG: GTG technique
LCRs: low copy repeats
LIS1: Lissencephaly 1
MDS: Miller - Dieker syndrome
MLPA: Multiple Ligation - dependent Probe Amplification
PAFAH1B1: Platelet- Activating Platelet-Activating Factor Acetylhydrolase, Isoform 1B
PGD: Preimplantation Genetic Diagnosis
PHA: Phytohemagglutinin
RAI1: Retinoic Acid -Inducible 1
RCTs: Reciprocal Chromosomal Translocations
SMS: Smith - Magenis syndrome
Th – L: Thoracic and lumbar section of the spine
TP53: Tumor protein gene
